# The same genomic variants in the first three exons of *KANSL1* can be either benign or causative of Koolen-de Vries syndrome: Definition of a validation procedure

**DOI:** 10.1016/j.gendis.2025.101546

**Published:** 2025-01-27

**Authors:** Federica Francesca L'Erario, Giuseppe Marangi, Anna Gloria Renzi, Marina Carapelle, Paolo Niccolò Doronzio, Domizia Pasquetti, Sabrina Maietta, Elena Sonnini, Annalisa Gazzellone, Marcella Zollino

**Affiliations:** a Section of Genomic Medicine, Department of Life Sciences and Public Health, Università Cattolica del Sacro Cuore, Rome 00168, Italy; b Unit of Medical Genetics, Department of Laboratory and Infectious Disease Sciences, Fondazione Policlinico Universitario A. Gemelli IRCCS, Rome 00168, Italy

Koolen-de Vries syndrome (KdVS, OMIM #610443) is a neurodevelopmental disorder characterized by distinctive facial characteristics, intellectual disability, and friendly behavior. A full KdVS phenotype can be caused by a recurrent 17q21.31 deletion of 0.3–0.6 Mb, as observed in about 70%–80% of cases, or by predicted truncating variants (PTVs) in *KANSL1* (KAT8 regulatory NSL complex subunit 1) in the remaining 20%–30% of patients.^1^^,^^2^ PTVs were reported to affect any coding exon of the gene from 2 to 15 in typical KdVS patients. However, polymorphic duplications including exons 1–3 of *KANSL1* (NM_015443.4) can make the final diagnosis of KdVS challenging (Suppl.Notes).^3^ Since certain PTVs in the first three exons of *KANSL1* were described either as pathogenic or benign, likely depending on whether they affect the functional or the polymorphic copy of the gene, a gene-specific strategy for validation is needed.

We describe six unrelated patients with variants in exons 1–3 of *KANSL1*, who were referred after a preliminary diagnosis of KdVS, in whom the pathogenicity of the *KANSL1* variants was reassessed based on both a deeper clinical re-evaluation and further laboratory analyses.

Genomic abnormalities consisted of apparent deletions of the first three exons of *KANSL1*, called by array-CGH, in two subjects (No. 71 and 72) and PTVs in exon 2 in the remaining four, including the NM_015443.4:c.985_986del variant in three subjects (No. 67, 68, and 69)^4^ and the NM_015443.4:c.908_909del variant in one (No. 70) (Fig. 1A). All these variants were detected by array-CGH or by next-generation sequencing gene panel analyses that were performed for unexplained intellectual disability.

**Figure 1:**
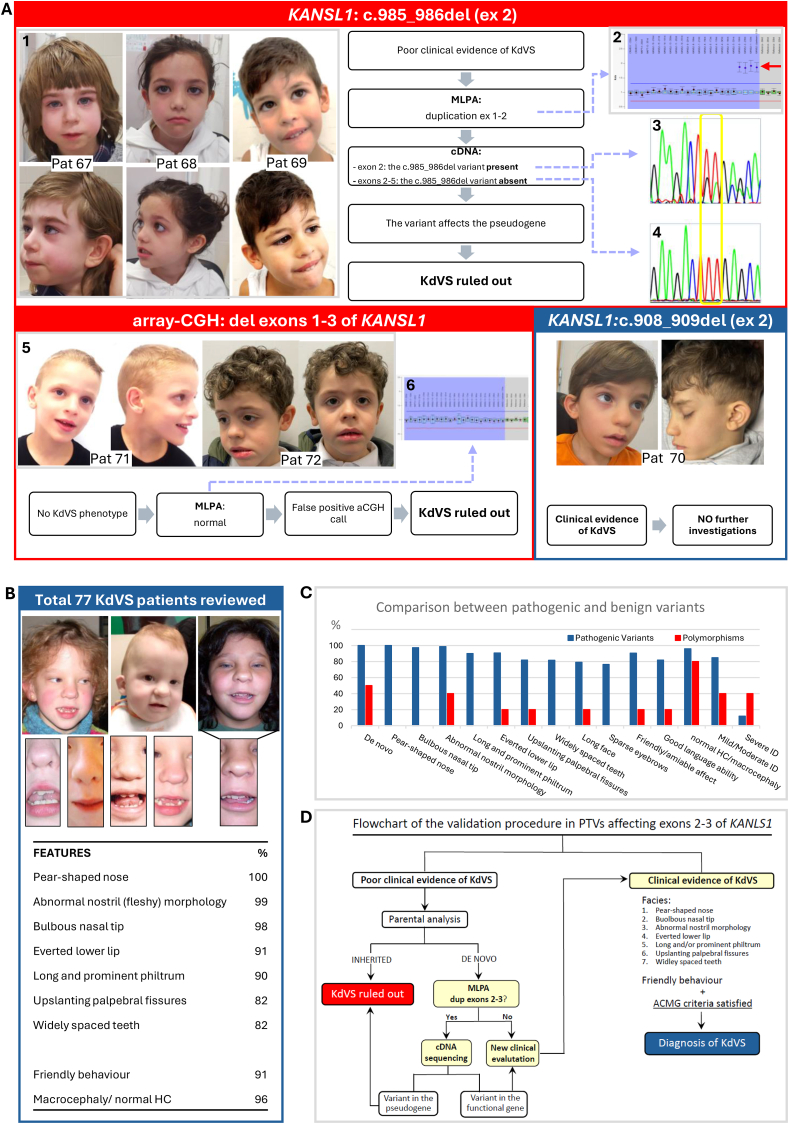
Schematic representation of the clinical and molecular findings that were considered for assessing the pathogenicity of variants in exons 1–3 of *KANSL1.***(A)** Top red panel: Clinical pictures and molecular data of patients 67,^4^ 68, and 69 with the c.985_986del variant on exon 2 of *KANSL1*. **(A1)** Frontal and lateral view of patients: except for mild upslanting of the palpebral fissures and prominent and long philtrum in patient 67, and of bulbous nasal tip in patient 68, all of them lack the full spectrum of the most distinctive facial features of KdVS. **(A2)** MLPA analysis: duplication of the first two exons of *KANSL1* was observed in all of them. **(A3)** cDNA sequencing with primers amplifying only exon 2: the c.985_986del variant diagnosed on genomic DNA was detected on cDNA as well. **(A4)** cDNA sequencing with amplicons spanning exons 2–6: the c.985_986del variant is absent. **(A)** Bottom red panel: clinical pictures and molecular data of patients 71 and 72 with exons 1–3 deletion called by array-CGH. **(A5)** Frontal and lateral view of patients: the typical features of KdVS are not appreciable in either. In patient 71, please note the presence of a high forehead; receding hairline on the forehead; large and deep-set eyes; large, medially flaring and sparse eyebrows; high nasal bridge with prominent columella; open mouth, pointed triangular chin and linearized mandibular bones. The clinical hypothesis of a mild presentation of Mowat-Wilson syndrome was confirmed by the detection of the c.3171_3172del variant in exon 10 of *ZEB2* (Suppl.P&M). **(A6)** MLPA analysis: no deletion of exons 1–3 was detected in either. This discrepancy was attributed to a false positive call in array-CGH analysis, contingent on the control samples used. **(A)** Bottom blue panel: clinical pictures of patient 70 with the *de novo* c.908_909del in exon 2 of *KANSL1*. **(A6)** Frontal and lateral view of the patient: the full spectrum of the most distinctive facial features of KdVS can be appreciated, including the typical nose and mouth conformation. No further molecular investigations were deemed necessary. **(B)** Revision of 77 KdVS patients (Table S1). Included in the present revision were both patients of personal observation and patients reported in the literature with detailed clinical information and pictures. The facial characteristics of some subjects (No. 26, 28, and 2 previously reported)^1^ and the specific nose, mouth, and teeth conformation are highlighted. Below the clinical pictures, a selected list of highly distinctive clinical signs in KdVS is shown, according to their frequency and specificity. (HC=head circumference) **(C)** Comparison between pathogenic and benign variants in *KANSL1*. The most frequent clinical manifestations resulting from the revision of 77 KdVS subjects (blue bars) are compared with those of our patients with benign variants in *KANSL1* (red bars). The additional features considered in this comparison, in particular sparse eyebrows, long face, good language ability, and the degree of intellectual disability, were not included among the most distinctive clinical signs shown in (B), since most of them are age-related. Please note that the *de novo* occurrence of variants is not sufficient in assessing their pathogenicity. (ID=intellectual disability) **(D)** Flowchart of the validation procedure in PTVs affecting exons 2 and 3 of *KANSL1*. The first step is clinical evaluation (or re-evaluation) of patients: whether the typical KdVS facial features, especially if associated with friendly behavior, can be appreciated, and the ACMG criteria for pathogenicity are satisfied, a definitive diagnosis of KdVS can be made without further molecular investigations (right side). In the absence of the full spectrum of the facial characteristics of KdVS, the inheritance of the detected variant has to be assessed by parental analysis. If the variant is inherited from a non-mosaic healthy parent, it is considered benign, and the diagnosis of KdVS can be ruled out. In cases of *de novo* PTVs, MLPA analysis of *KANSL1* is indicated. If a polymorphic duplication of exons 1–3 of the gene is detected, cDNA sequencing of different amplicons, as illustrated in (A3) and (A4), is indicated, to assess the localization of the variant within either the functional copy of the gene or within its nonfunctional duplicated segment. Whether, by MLPA, a duplication polymorphism in *KANSL1* is not detected, and whether, by cDNA sequencing of amplicons spanning from exon 2 to other exons not involved in the duplication polymorphism, the variant can be inferred to affect the functional gene, the expected clinical phenotype is KdVS, and a conscious clinical re-evaluation is recommended, following the here highlighted stringent clinical criteria. Deletions of exons 1–3 of *KANSL1* are not considered in the present flowchart. Actually, to date they have never been described in typical KdVS subjects, leading to the conclusion that they are false positive calls when detected in array-CGH analysis, most likely. However, *KANSL1* MLPA analysis can be performed to support this conclusion.

We found that the clinical phenotype was fully consistent with KdVS in only one subject (No. 70; c.908_909del). Being the genomic results deemed diagnostic, no further molecular investigations were planned. On the contrary, although some signs could be consistent with KdVS, on deeper clinical analysis, the diagnosis of KdVS was questionable in all the remaining five subjects (Suppl.P&M). They were then enrolled into multiplex ligation-dependent probe amplification (MLPA) and/or cDNA sequencing of *KANSL1*. Regarding the two subjects with apparent exons 1–3 deletion of *KANSL1* (No. 71 and 72), that was *de novo* in one, MLPA gave normal results (Fig. 1A6), leading to attribute this discrepancy to a common false positive call in array-CGH analysis, contingent on the control samples used. The diagnosis of KdVS was definitively ruled out in both. In addition, a milder presentation of Mowat-Wilson syndrome was considered in one subject (No. 71) and confirmed by the detection of a *de novo* frameshift variant in exon 10 of *ZEB2* (zinc finger E-box binding homeobox 2) (Suppl.P&M). Regarding the three subjects with the c.985_986del variant in exon 2 (No. 67, 68, and 69), which was *de novo* in one, we performed *KANSL1*-specific MLPA (Suppl.P&M), which detected the polymorphic duplication of exons 1 and 2 on genomic DNA in all of them (Fig. 1A2). Then, we performed a cDNA analysis of *KANSL1*. To assess whether the variant affected the functional copy of the gene or the highlighted duplicated segment, different amplicons were simultaneously analyzed in individual patients, namely amplicons limited to exon 2 and amplicons spanning from exon 2 to other non-duplicated exons. The variant could be observed only in amplicons limited to exon 2 (Fig. 1A3, 4). We concluded that the c.985_986del variant in these cases affected the non-functional copy of the gene, and it was defined as benign. The diagnosis of KdVS was definitively ruled out in all these subjects.

Thus, variants in the first three exons of *KANSL1* deserve specific care in their interpretation, since they are included in a region known to be subject to structural polymorphisms (Suppl.Notes). More precisely, a duplication of a genomic segment that includes exons 1–2 or 1–3 of *KANSL1* can be found in more than 40% of alleles in European populations.^4^ To the best of our knowledge, such a duplicated fragment of the gene is non-functional, but it may constitute a cause of misdiagnosis in routine genetic testing.

The validation procedure we suggest is driven primarily by the specific evaluation of KdVS-associated clinical traits in patients. To highlight the most distinctive clinical features of KdVS, we reviewed a total of 77 KdVS individuals (Suppl.PM Table S1). Highly specific for KdVS were the facial characteristics, including upslanting of palpebral fissures (82%), pear-shaped nose with flashy nares and bulbous tip (almost 100%), long and/or prominent philtrum (90%), everted lower lip (91%), widely spaced teeth (82%), friendly behavior (91%), and normal head circumference/macrocephaly (96%) (Fig. 1B; Suppl.P&M). When comparatively evaluated, they allowed for the effective distinction between KdVS and non-KdVS patients with variants in *KANSL1* in our cohort (Fig. 1C).

All the facial features and the friendly behavior are preliminarily suggested as major diagnostic criteria for KdVS (Fig. 1D). Supportive clinical criteria could include marked speech delay highly responsive to speech therapies, abnormal hair/color texture, and failure to thrive in infancy. These preliminary suggestions will hopefully provide the basis for the definition of a clinical score system, on a multicenter collaborative approach. Whenever the clinical phenotype appears fully consistent with KdVS, no further investigations are needed.

Following a clinically based hypothesis of a misdiagnosis of KdVS, a different scenario is to be considered. In cases of apparent deletions of exons 1–3 of *KANSL1*, called by array-CGH, further investigations are not mandatory, and they can be interpreted as false positive calls in array-CGH analysis. However, *KANSL1* MLPA analysis, expected to give normal results, can support this conclusion.

In cases of apparent PTVs, the second step in the validation procedure we suggest is *KANSL1* MLPA analysis on genomic DNA first, to search for the presence of a duplication polymorphism in patients, and then cDNA sequencing of both amplicons limited to exon 2 or 3 (depending on where the variant is located) and amplicons spanning from exon 2 to exons not affected by the common polymorphic duplications. If the PTVs are limited to the duplicated non-functional copy of the gene, the diagnosis of KdVS can be definitively ruled out.

Some other considerations are in order. In two patients initially diagnosed with KdVS, the c.985_986del variant in *KANSL1* was inherited from the healthy father. Defective penetrance was tentatively considered to underlie this parent-to-child discrepancy. However, pathogenic variants in *KANSL1* are fully penetrant, leading to the conclusion that the detection of constitutional PTVs in one healthy parent is sufficient to rule out the diagnosis of KdVS. At the same time, the *de novo* occurrence of variants cannot allow for a definite assessment of pathogenicity, as we observed in a subject in the present series.

We wondered whether the c.985_986del variant should be defined as benign on any occasion (Suppl.Notes). We found that it was described to cause typical KdVS.^2^ Thus, defining the nature of any PTV affecting exons 1–3 of *KANSL1* is not the variant itself, but rather its genomic localization. A search for the KdVS-specific DNA methylation signature can help the assessment of the pathogenicity of variants in the first three exons.^5^

In conclusion, the classification of variants affecting the first three exons of *KANSL1* demands a specific approach due to the unique genomic architecture of the 17q21.31 region (Fig. 1D). The validation procedure we suggest is mainly driven by clinical genetics, and reinforced by easily available molecular investigations, namely MLPA and cDNA sequencing of *KANSL1*.

## Ethics declaration

The study was conducted in accordance with the Declaration of Helsinki and approved by the Institutional Review Board of the Department of Life Sciences and Public Health of the Catholic University of Roma, Italy (Prot. No. DIPUSVSP-PD-07-242). Parental consent was obtained.

## Funding

This work was supported by PRIN (Progetto di Ricerca di Rilevante Interesse Nazionale) 2022 (No. L4F87B to M.Z.) funded by the European Union NextGenerationEU and the Italian "Ministero dell’Università e della Ricerca".

## CRediT authorship contribution statement

**Federica Francesca L'Erario:** Writing – original draft, Investigation, Data curation. **Giuseppe Marangi:** Writing – original draft, Software, Formal analysis. **Anna Gloria Renzi:** Investigation, Formal analysis. **Marina Carapelle:** Validation, Formal analysis, Data curation. **Paolo Niccolò Doronzio:** Software, Resources, Formal analysis. **Domizia Pasquetti:** Writing – original draft, Methodology, Data curation. **Sabrina Maietta:** Investigation, Formal analysis. **Elena Sonnini:** Resources, Investigation, Data curation. **Annalisa Gazzellone:** Resources, Investigation, Data curation. **Marcella Zollino:** Writing – review & editing, Validation, Supervision, Methodology, Funding acquisition, Conceptualization.

## Conflict of interests

The authors declared no conflict of interests.
