# Supplementary material for: The same genomic variants in the first three exons of KANSL1 can be either benign or causative of Koolen-de Vries syndrome: Definition of a validation procedure
Source: Genes Dis. 2025 Jan 27;12(6):101546. doi: 10.1016/j.gendis.2025.101546 (PMC12304670; doi:10.1016/j.gendis.2025.101546)
Supplement: Multimedia component 1 [file mmc1.docx]

**Supplementary NOTES**

**Genomic architecture and haplotypes within chromosome region 17q21.31**

Approximately 5% of the human genome is composed of segmental duplications (also known as duplicons or low copy repeats, LCR) that can act as substrates for non-allelic homologous recombination (NAHR).^6^ NAHR is a major cause of chromosomal rearrangements including deletions, duplications, or inversion, with the majority of these being clinically benign. The recurrent 17q21.31 deletion in KdVS syndrome, spanning on average 0.5-0.6 Mb, is caused by NAHR between two surrounding LCRs. The same duplicons are at the origin of a clinically harmless 900 kb inversion polymorphism, defining the so called H2 haplotype, which shows an allele frequency of about 20% in European populations.^3,7,8^

Segmental duplications are copy number polymorphic and they can also contain transcriptionally active genes. One of the LCRs in the 17q21.31 region includes the promoter and the first three exons of the *KANSL1* gene (**exon numbering is referred to the MANE Select transcript NM_015443.4**), roughly spanning between chromosome 17 positions 46090 kb and 46220 kb, as to referred to the human genome assembly GRCh38. It is duplicated in different haplotypes giving rise to novel *KANSL1* transcripts that include the promoter and the first three or two exons, when associated with either the H1 or the H2 haplotypes, respectively. Almost 60% of Europeans carry at least one of these duplications (see Supplementary Figure for a schematic representation of the structural forms of the most frequent 17q21.31 haplotypes in populations of European descent).

To the best of our knowledge, the transcript from the duplication associated with the partial pseudogene of *KANSL1* code for no functional protein, even though it appears to be expressed.


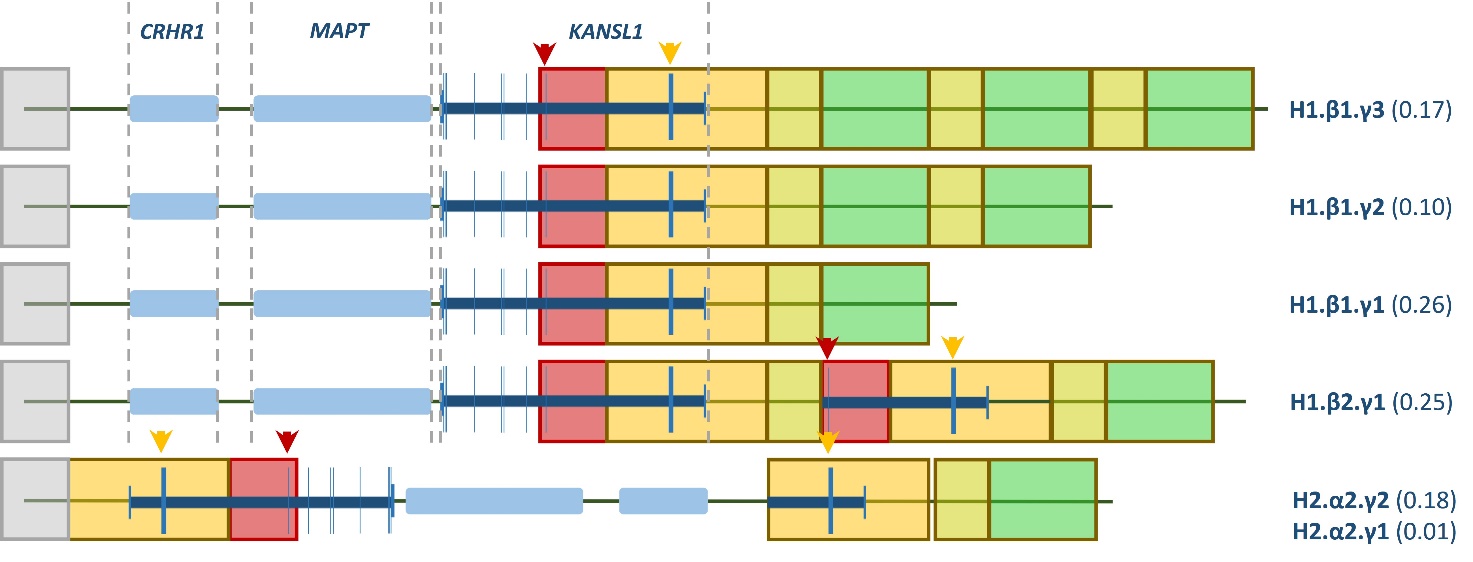


**Supplementary Figure 1:** Structural forms of the most frequent 17q21.31 haplotypes in populations of European descent (according to Boettger et al. 2012).^3^ In brackets: haplotype frequencies in CEU population. Only CRHR1, MAPT (pale blue rectangles) and KANSL1 (with exons represented as vertical bars) are depicted. Colored boxes stand for different segmental duplication blocks. Yellow arrowheads point to regions containing the sequence of KANSL1 exon 2 (NM_015443.4). Red arrowheads point to regions containing the sequence of KANSL1 exon 3.

It must be stressed that only the duplication is polymorphic and benign, while the corresponding deletion of the first two or three exons of the functional copy of the *KANSL1* would necessarily cause the Koolen-de Vries syndrome.

Indeed, such deletions are reported in the Database of Genomic Variants (http://dgv.tcag.ca/dgv/app/home) to have been detected in apparently healthy individuals. This finding is likely explained by the fact that some studies relied on chromosomal microarrays based on comparative genomic hybridization using control samples that might have the duplication polymorphism.

Thus, deletions of the region containing the first three exons of *KANSL1* (but none of the others) detected by array-CGH analysis (and similar methods) need to be carefully evaluated, since they are pathogenic if they are real, but, in most cases, they are just false positive results due to the limits of the technique.

Nonetheless, partial *KANSL1* deletions limited to the first 3 exons of the *KANSL1* gene have never been described in typical KdVS patients, with the sole exception of a report defined as pathogenic in ClinVar (VCV000978035.3) for which, however, no clinical information is available. Whether the deletion of exons 1-3 of *KANSL1* detected by array-CGH is always to be considered a benign polymorphism cannot be stated exactly. MLPA is recommended in these cases, searching for confirmation of the deletion. However, being this rearrangement unusual in KdVS, a deep clinical re-evaluation of patients is recommended also in case of confirmation.

*KANSL1*-specific MLPA allows for a more precise characterization of control samples and, consequently, for the proper distinction between subjects with the duplication polymorphism, the non-duplicated allele and the pathogenic partial gene deletion.

It must be also considered that normal MLPA results (i.e.: biallelic status for all the exons of the gene) might underlie a compound heterozygosity for a benign duplication polymorphism and a pathogenic deletion of the first two exons in unique patients, even though it is a quite unlikely scenario, considering the rarity of partial *KANSL1* deletion reported so far and the fact that it should be limited to the first two or three exons at most.

The duplication polymorphism containing the *KANSL1* pseudogene poses relevant issues also for the interpretation of sequence variants called within the *KANSL1* gene. *KANSL1* gene and pseudogene sequences share very high sequence homology, that does not allow for the distinction between them, neither with short-read massive parallel sequencing methods, nor with Sanger sequencing. Long-read sequencing that can resolve complex structural variant could, in theory, allow for the correct alignment and annotation.

Such regions of high sequency homology constitute a well-known issue hampering the accuracy of many diagnostic tests in genetics. It has been estimated that 619 genes, of which 73 are clinically relevant, contain large contiguous regions (including one or more exons) that prevent them to be univocally mapped in short-read massive parallel sequencing. These numbers are only slightly reduced for standard Sanger sequencing approach (467 genes, of which 54 clinically relevant), while they are four to five times higher when considering also potentially difficult sites (2512 genes, 464 clinically relevant).^9^

It must be highlighted that sequence variants called in the first three exons of *KANSL1*, in individual with the duplication polymorphism, may appear as mosaic, since more than two copies of the regions are present.

Even though we deal with non-synonymous in the coding portion of the *KANSL1* gene (i.e.: in exons 2 and 3), exon 1 of the transcript NM_015443.4 (and any other alternative 5’ exon of *KANSL1* upstream the first protein coding exon) belongs entirely to the 5’UTR and variants involving that region, or the upstream promoter, could be responsible for Koolen-de Vries syndrome, thus the suggestions we propose apply to exon 1 as well, at least in theory.

The strategy we propose to establish if a variant affect the functional *KANSL1* gene or its pseudogene implies the analysis of *KANSL1* gene and pseudogene transcripts by using different combination of primers, since the pseudogene appears to be expresses as well, but primers targeting sequences in exons from the 4^th^ to the last are specific for the full *KANSL1* mRNA.

On the other hand, to the best of our knowledge and experience, duplications involving exons 4 to 15 of the *KANSL1* gene are not usual laboratory findings, thus they are not to be considered polymorphic by definition and they should be assessed on an individual basis. Even though in the “Database of Genomic Variants” there are some such duplications reported, their rarity suggests caution in the pathogenicity classification. In any case, MLPA analysis provides a relevant support for the evaluation of *KANSL1* variants in general. In this paper, we mainly focus on polymorphic duplications involving exons 1-3 since their frequency may represent a major issue in clinical settings.

**Population *KANSL1* variants**

In the gnomAD database (https://gnomad.broadinstitute.org/), considering the latest version (v4.1.0), 30 predicted loss-of-function (pLoF) variants in the second exon of the MANE Select transcript (NM_015443.4) are reported in a heterozygous state in a total of 155 subjects out of 807,162. For instance, the c.985_986del we describe on is reported with a frequency of 0.000005085 in non-Finnish European population (6 out of 1180044 alleles) and of 0.0002702 (8 out of 29606 alleles) in Ashkenazi Jewish population. We expect that the vast majority of those variants, especially the recurring ones, are actually mismapped and involve the polymorphic duplication of *KANSL1* exon 2. Supporting this hypothesis, available read data show a variant/reference read ratio around one third (or less) for most of them, consistent with the involvement of a duplicated region. Notably, two gnomAD variants within exon 2 have been classified as “Pathogenic” in the ClinVar database (https://www.ncbi.nlm.nih.gov/clinvar/), in accordance with the fact that such variants would be detrimental if they involve either functional copy of the KANSL1 gene.

Two further pLoF variants (in two heterozygous individuals) are reported in exon 3, which may be involved in the duplication polymorphism in some haplotypes. Population variants in the remaining exons deserve a separate discussion. PTVs in the last exon are not expected to trigger the nonsense-mediated decay (NMD) a some of them might be benign (i.e.: the NM_015443.4:c.3312_3313del shows a MAF of about 0.1 % in East Asian population, which is scarcely consistent with KdVS prevalence). Variants reported in exons from 4 to 14 in the gnomAD database must be taken with a grain of salt, especially singletons, since some might have been found in individuals who are indeed unacknowledged patients with KdVS (quite unlikely though), some might be false positive calls, and some other might underlie specific molecular mechanisms (i.e.: two reportedly distinct frameshift variant that are actually in cis in the same allele thus restoring the standard reading frame, or variants affecting an alternative acceptor splice site upstream exon 11).

**KdVS-specific DNA methylation signature**

Recently, a unique DNA methylation signature (episignature) has been specifically generated and validated for KdVS.^5^ KdVS-specific episignature evaluates the molecular consequences of KANSL1-haploinsufficiency: in case of positive results (which are usually associated with a consistent phenotype) we might expect that a certain variant in *KANSL1* is pathogenic, in case of negative results (and no KdVS phenotype) a *KANSL1* variant is not likely to be responsible for KdVS. In the published study, it allowed for the correct classification of a series of loss-of-function variants in *KANSL1* as responsible for KdVS, including three protein-truncating variants (identified in four different patients) involving exon 2. Notably, among them, the variant NM_015443.4:c.808_809del(p.Leu270ValfsTer11) has been reported in 26 individuals in the gnomAD database (https://gnomad.broadinstitute.org/) with a frequency of 0.00001611, which in usual diagnostic settings would have led the variant to be deemed as benign.

This observation further supports the point that specific strategies are required for the proper classification of variants claimed to affect exons from 1 to 3 of *KANSL1*, since they may be either pathogenic or benign, based on the involvement of either the pseudogene or the functional allele of *KANSL1*.

In the same study, another variant in the second exon, specifically the NM_015443.4:c.727C>T (p.Gln243Ter), was not accompanied with the KdVS-associated episignature, in accordance with the patient’s clinical features not resembling the KdVS phenotype.

Thus, the analysis of syndrome-specific episignatures constitutes an efficient method for variant pathogenicity evaluation that can be applied also to variants in exons 1-3 of *KANSL1*, when the method is available for the testing laboratory. Episignature should be used as supporting information for variant evaluation, providing further clues that a variant in the first three exons involves either coding genes or pseudogenes. However, for a definitive confirmation, cDNA analysis is required, since episignature provides no direct information about the structure of the genomic region.

**References**

1. She X, Jiang Z, Clark RA, et al. Shotgun sequence assembly and recent segmental duplications within the human genome. *Nature*. 2004;431:927–930.
2. Steinberg KM, Antonacci F, Sudmant PH, et al. Structural diversity and African origin of the 17q21.31 inversion polymorphism. *Nat Genet*. 2012;44:872–880.
3. Stefansson H, Helgason A, Thorleifsson G, et al. A common inversion under selection in Europeans. *Nat Genet*. 2005;37:129–137.
4. Mandelker D, Marra A, Zheng-Lin B, et al. Genomic Profiling Reveals Germline Predisposition and Homologous Recombination Deficiency in Pancreatic Acinar Cell Carcinoma. JCO. 2023;41:5151–5162.
