# Supplementary material for: The same genomic variants in the first three exons of KANSL1 can be either benign or causative of Koolen-de Vries syndrome: Definition of a validation procedure
Source: Genes Dis. 2025 Jan 27;12(6):101546. doi: 10.1016/j.gendis.2025.101546 (PMC12304670; doi:10.1016/j.gendis.2025.101546)
Supplement: Multimedia component 2 [file mmc2.docx]

**Supllemetary patients and methods**

**Case series**

A total of six patients with ID and physical were referred for a second opinion after a preliminary diagnosis of KdVS. Apart from patient 67 described in Bigoni and colleagues’ report^4^ (Fig.1-A1), they were never described before.

Subject 68. This is a 9-year-old girl of Italian ancestry, born as the first child to healthy and non-consanguineous parents by normal delivery. Pregnancy was uneventful. Birth weight (BW) was 4080 g (90^th^ – 97^th^ centile), and length (L) 52 cm (75^th^ – 90^th^ centile). She was born with cleft palate, which was surgically resolved. No hypotonia was noticed from early on. Motor milestones were normal, she walked unsupported at 14 months. She spoke her first words at 12 months. At age 5 years, she experienced the first episode of seizures, characterized by stiffness, deviation of the oral commissure to the left, and loss of consciousness for approximately 2 minutes. The frequency of seizures increased up to around 15 absence episodes per day. Moreover, EEG examination revealed frequent spikes and waves in the temporal regions and paroxysmal activity in the frontotemporal region on the left. On brain MRI, reduced perfusion of the basal frontal, opercular frontal, and temporal regions was detected, with no structural anomalies. A therapy with sodium valproate and ethosuximide was initiated, successfully. Till now, the patient has not experienced further episodes of seizures. She was diagnosed with mild/borderline ID. Elsewhere, a diagnosis of KdVS was made following the identification of the c.985_986del variant in exon 2 of *KANSL1* (NM_015443.4) (NC_000017.11:g.46171159_46171160del) (ClinVar variant accession No.: VCV000038930.6, submission accession No.:SCV005061832.1) by NGS analysis of a large gene panel for neurodevelopmental disorders. The variant was absent in both parents.

When we first saw her at age 9 years, weight (W) was 24,2 kg (10^th^-25^th^ centile), height (H) 129 cm (50^th^-75^th^ centile) and occipital frontal circumference (OFC) 53 cm (40^th^ centile). A sociable behaviour was described, even if generally oppositional. She was able to pronounce many sentences. Some peculiar facial characteristics were noted, including bilateral epicanthal folds, short nose with depressed nasal bridge and bulbous tip, prominence of the lower lip (Fig.1-A1). She presented with ligamentous hyperlaxity.

Some clinical signs, particularly the bulbous tip of the nose, the prominent lower lip and the friendly behaviour, were consistent with KdVS. However, at deeper clinical evaluation, the diagnosis of KdVS appeared to be questionable, and she was enrolled into MLPA and cDNA sequencing of *KANSL1*.

By MLPA, the polymorphic duplication that includes exons 1 and 2 of KANSL1 was detected on genomic DNA (Fig.1-A2). With cDNA sequencing, the c.985_986delTT variant could not be observed in amplicons spanning from exon 2 to exon 4 and from exon 2 to exon 6, while it was observed in amplicons limited to exon 2 (Fig.1-A3,4). The electropherogram showed that the height of peaks corresponding to the variant transcript were, on average, one third of those corresponding to the standard one, supporting the hypothesis that transcripts from three different alleles are captured: two from the functional copies of the *KANSL1* gene and one from the pseudogene (which corresponds of the duplicated first two exons of *KANSL1*) assuming that they are expressed at similar amounts. These results may be explained by the scenario in which the variant involves the duplicated non-functional pseudogene, which is actively expressed and does not undergo nonsense-mediated decay (NMD).

Subject 69. He is a 4 ^7/12^ old male, the only child of healthy non-consanguineous parents. He was born at 41 weeks by normal delivery. BW was 3150 g (15^th^ centile), L 50 cm (37^th^ centile), and OFC 35.5 cm (75^th^ centile). Apgar scores were 8 and 9 at 1’ and 5’, respectively. Pregnancy was complicated by gestational diabetes. On first-trimester foetal ultrasound, a suspicion of cerebellar hypoplasia was raised, but it was ruled out later. At birth, he was diagnosed with hypotonia. Motor milestones were delayed, he walked unsupported at age 21 months, gait was unstable. He never said any words. ID was moderate to severe, associated with behaviour abnormalities, including hyperactivity and irritability. Brain MRI at age 3 years revealed a widespread cerebellar dysplasia, affecting cerebellar hemispheres and the vermis, which was malformative in nature, most likely. Elsewhere, he first underwent CMA, with normal results, and then NGS sequencing of a panel of 370 genes associated with brain malformation, that allowed for detection of the c.985_986del variant (NM_015443.49) in exon 2 of *KANSL1*. A diagnosis of KdVS was made. Being the same variant inherited from the healthy father, a hidden mosaicism or defective penetrance was hypothesized in the father.

When we first saw him at age 4 ^7/12^, W was 14 kg (3^th^ centile), L 96 cm (3^th^ centile), OFC 49.5 cm (-2.2 SD). Peculiar facial characteristics were noted, including synophrys, megalocornea, bilateral epicanthus, depressed nasal bridge, full lips, short philtrum, protruding ears with flattened helices in the 3^rd^ superior (Fig.1-A1). He also presented with tapered fingers, clinodactyly of the fifth finger, bilaterally, and a small sacral angioma.

The diagnosis of KdVS was unlikely, clinically. To support this conclusion, both the patient and the father underwent MLPA and cDNA sequencing of *KANSL1*. MLPA showed that the polymorphic duplication including exons 1 and 2 of KANSL1 was detected on genomic DNA from both the patient and his father. The results of cDNA sequencing analysis were similar to those obtained for patient 68 (Fig.1-A2,3,4).

Subject 70. This is a 3-year-old male, the only child of healthy non-consanguineous parents, born at 39 weeks by caesarean section due to foetal distress. Growth restriction was noted since the 28^th^ week of gestation. BW was 2760 g (10-25^th^ centile), L 48 cm (50-75^th^ centile), OFC 33 cm (30^th^ centile). Ventricular asymmetry with dilatation of the anterior horn was detected at birth by brain ultrasound examination. On the second day of life, he experienced several episodes of seizures, that were promptly controlled by anticonvulsant therapy. Single episodes of febrile seizures occurred at 9 and 29 months of life, and at age 3 years focal seizures were observed. Mild dilation of the renal pelvis, atrial septal defect (ostium secundum), and patent foramen ovale (PFO) were detected on ultrasound examinations. He presented with global neurodevelopmental delay. He walked unsupported at 24 months, at age 3 years language was absent. Additional clinical signs included conductive unilateral hearing loss and recurrent pulmonary infections.

He was referred to us with a diagnosis of KdVS caused by the variant NM_015443.4: c.908_909del, p.(Lys303Thrfs*11) (NC_000017.11:g.46171235_46171236del) (ClinVar variant accession No.: VCV003250486.1, submission accession No.:SCV005061833.1) in exon 2 of the *KANSL1* gene, that was absent in both parents. No further anomalies were detected with MLPA analysis, including the duplication polymorphism. At our first clinical evaluation at the age 3 years, W was 10.5 kg (-2.2 SD), H 88 cm (10^th^ percentile), and OFC 48.8 cm (25^th^ percentile). A lovely behaviour was referred from early on. Distinctive facial features were observed, including pear-shaped nose with bulbous tip and fleshy nares, long philtrum, protruding and thickened lower mucosal border, spaced teeth, prognathism, and large cup-shaped ears (Fig.1-A, blue panel). An unequivocal diagnosis of KdVS was made, clinically.

Subject 71. This is a 4 ^8/12^ male, the only child of healthy non-consanguineous parents, born at 36+4 weeks by caesarean section due to gestational diabetes. BW was 2370 g (13^th^ centile). Double renal pelvis on the left was detected prenatally. At birth, patent ductus arteriosus (PDA) and patent foramen ovale (PFO) were diagnosed, corrected by percutaneous closure at 12 months. He started walking at 18 months and spoke his first few words at 16 months. Language is currently poor, limited to about 15 single words. The reason for referral was a de novo heterozygous deletion on 17q21.31 detected by means of array-based comparative genomic hybridization (array-CGH) encompassing the first three exons of *KANSL1* (arr[GRCh38]17q21.31(46094522-46219777)x1), leading to consider a diagnosis of KdVS. When we first saw him, at age 4 ^8/12^ years, W was 19 kg (50-75^th^ centile), H 109 cm (50^th^ centile), OFC 50.8 cm (25^th^-50^th^ centile). Some distinctive facial features were noticed, including sparse eyebrows, long nose with a prominent septum, thick lower lip, slim and long face, linearization of the mandibular angle, large and normally shaped ears (Fig.1-A5). He also had short hands with widened terminal phalanges, and unilateral cryptorchidism.

MLPA of *KANSL1* gave normal results (Fig.1-A6), leading to conclude that causing a misdiagnosis, by array-CGH, of exons 1-3 deletion of *KANSL1* was the presence of the polymorphic duplication in the control DNA.

We found the clinical phenotype not consistent with KdVS, but rather with a milder presentation of Mowat-Wilson syndrome (MWS). That prompted us to perform *ZEB2* sequencing, and a de novo frameshift variant NM_014795.4:c.3171_3172del,p.(Cys1057*) (NC_000002.12:g.144389927_144389928del) (ClinVar variant accession No.: VCV003250487.1, submission accession No.:SCV005061831.1) in the last coding exon (i.e.: 10) of ZEB2 was identified at a heterozygous status. As expected from its location, and confirmed by the clinical phenotype, this variant was defined as hypomorphic, similarly to other variants already reported in the last exon of ZEB2.^10^

Subject 72. This is a 6-year-old boy, born at born at 36 weeks + 6. Biological parents are not available. BW was 3010 g (60^th^ centile), L 50 cm (90^th^ centile) and OFC 34.5 cm (90^th^ percentile). Motor milestones were slightly delayed. Main clinical manifestations were in the spectrum of behaviour abnormalities, including sleep disturbances, tendency to bring objects into the mouth, hyperactivity and poor interaction with the environment. Language was absent, no episodes of either auto- and hetero-aggressiveness were referred. The reason for referral was the detection, by array-CGH, of a 17q21.31 deletion limited to exons 1-3 of *KANSL1* (arr[GRCh38]17q21.31(46082437-46267672)x1). This rearrangement was considered either causative of KdVS or a benign polymorphism by two independent clinicians who evaluated him elsewhere. At age 6 years, when we first saw him, W was 18 kg (10-25^th^ centile), H 113 cm (25-50^th^ centile), and OFC 51 cm (50^th^ centile). Some peculiar facial characteristics were noted, including synophrys, arched eyebrows, downslanting palpebral fissures, mild proptosis, short nose with anteverted nostrils and wide mouth (Fig-1-A5). He also presented with clinodactyly of the fifth finger, bilaterally, and about ten cutaneous nevi on the trunk. The diagnosis of KdVS was unlikely, clinically. He underwent MLPA of *KANSL1*, with normal results (Fig.1-A6), leading us to raise the same considerations as in subject 71. He was enrolled into WES analysis, a priority analysis of genes for RASopathies and chromatinopathies was planned.

Exome sequencing on the familial trios of patients 68 and 69 is ongoing as well.

**Methods**

Only the analyses we performed in our laboratory are described, since we do not have sufficient details about the genetic tests conducted on the reported patients before they were referred to our Center for further evaluation.

**Analyses on genomic DNA**

Multiplex Ligation-dependent Probe Amplification analysis (MLPA) with the kit P443-A2 *KANSL1* (MRC Holland, Amsterdam, Netherlands), using as references DNA samples from healthy subjects who had been previously ascertained not to carry the common duplication polymorphisms. DNA samples from KdVS patients with the recurrent pathogenic 17p21.31 deletion were used as positive controls. The analysis was carried out by standard procedures, according to the manufacturer’s instructions. Fragment separation was performed by capillary electrophoresis on a SeqStudio 8 Flex Genetic Analyzer (Thermo Fisher Scientific, Waltham, MA, USA) and the results were analysed using Coffalyser.net (MRC Holland, Amsterdam, Netherlands).

*ZEB2* analysis. The *ZEB2* status was investigated by HTS of a panel of 11 genes associated with Rett-like conditions (*TCF4*, *CNTNAP2, NRXN1, UBE3A, SLC9A6, MECP2, CDKL5, FOXG1, MEF2C, ZEB2, ATRX*), by using the Ion AmpliSeq Library Kit Plus (Thermo Fisher Scientific, Waltham, MA, USA) for library preparation, following manufacturer’s instructions, the Ion Chef instrument (Thermo Fisher Scientific, Waltham, MA, USA) for emulsion PCR, the Ion PGM (Thermo Fisher Scientific, Waltham, MA, USA) for sequencing and the Ion Reporter software (Thermo Fisher Scientific, Waltham, MA, USA) for variant calling . ANNOVAR command-line software was used for variant annotation.^11^

**Analyses on cDNA of *KANSL1***

Total RNA was obtained from short-term PHA-stimulated lymphocyte cultures established from patients’ heparinized blood samples, using the guanidinium thiocyanate–phenol–chloroform extraction method on both untreated and puromycin-treated cells, following standard procedures.

cDNA was obtained by reverse transcription of RNA (High-Capacity cDNA Reverse Transcription Kit, Thermo Fisher Scientific, Waltham, MA, USA). We searched for biallelic expression of *KANSL1* by cDNA sequencing. Primers used throughout all phases of the experimental study were designed using the NM_015443.4/ ENST00000432791.7 reference sequence from the GENECODE/Ensembl database (https://www.ensembl.org/index.html). Primers were designed utilizing the Primer3Input software tool (https://primer3.ut.ee/) and then validated with the UCSC In Silico PCR tool (https://genome.ucsc.edu/cgi-bin/hgPcr) and the OligoCalc tool (http://biotools.nubic.northwestern.edu/OligoCalc.html). Primers were synthesized on service by Merck KGaA/Sigma Aldrich (Primers and experimental conditions are available upon request).

Different combinations of forward and reverse primers were used to obtain either of the following:

1. amplicons limited to exon 2 and/or 3, that are hypothesized to be derived from both the functional whole length *KANSL1* gene and the duplicated fragment containing only the promoter region and the first 2 or 3 exons;
2. amplicons starting from exon 2 and ending in some of the exons that are not included in the common duplication polymorphisms (specifically 4, 5, 6 and 9). They should be representative only of those transcripts that derive from the functional copy of *KANSL1*.

Primer sequences are available upon request.

**Review of KdVS patients**

We performed a clinical-genetic revision of a total of 77 KdVS patients with either 17q21.31 deletions or intragenic loss-of-function variants in the *KANSL1* gene, of whom 55 were already reported^1,2,12-16^ and 20 are novel personal observations. With respect to patients reported in the literature, those with available extensive clinical description and pictures are included in the present review.

Among the total 77 subjects we reviewed with a molecularly confirmed diagnosis of KdVS diagnosis, evaluated clinical signs were: failure to thrive in infancy; relative/true macrochephaly or normal head circumference or microchephaly; developmental delay; ID (mild or moderate or severe); good language ability (after 5 years); friendly/amiable behaviour; presence on the face of: sparse eyebrows, long face, upslanting palpebral fissures, abnormal nostril morphology (namely fleshy nares), pear-shaped nose, bulbous nasal tip, long and/or prominent philtrum, everted lower lip, abnormal hair/colour texture and widely spaced teeth; ectodermal abnormalities (hyperpigmentation/ hypopigmentation, hyperkeratosis, dry skin/ eczema and oligodontia) and hearing impairment.

All signs recorded in individual patients are shown in Table S1.

However, highly distinctive clinical features, focused mainly on the facial characteristics, but also including the degree of ID, the growth pattern of head circumference and behaviour, are selected here to define the first and linear tool for the here discussed validation procedure*.*

KdVS patients had ID 57/59 (97%), that was mild in 25 (42%), moderate in 27 (46%), and severe in 7 (12%); upslanting palpebral fissures 63/77 (82%), sparse eyebrows 55/72 (76%), pear shaped nose 76/76 (100%), bulbous nasal tip 75/77 (97%), fleshy nares 76/77 (99%), long and/or prominent philtrum 63/70 (90%), fleshy everted lower lip 69/76 (91%), widely spaced teeth 40/49 (82%) relative macrocephaly 32/72 (44%), regular OFC according to height 37/72 (51%), microcephaly 3/72 (4%); and friendly behaviour 58/64 (91%). Thus, 95% of patients presented with normal head circumference or relative macrocephaly, and 88% with mild or moderate ID.

All the above mentioned signs are suggested as major diagnostic criteria for KdVS. Additional signs, including marked speech delay highly responsive to speech therapies; abnormal hair/colour texture; failure to thrive in infancy and short stature in adulthood are tentatively defined as supportive diagnostic criteria.

The same criteria were evaluated in our five subjects with a questionable KdVS phenotype in whom variants were identified apparently involving the first three exons of *KANSL1*, providing further evidence that they all received a misdiagnosis of KdVS. The histogram in Fig.1C shows the comparison between the relative frequencies of clinical features observed in these two categories of patients: those with confirmed KdVS and those with non-pathogenic variants in the duplicated region corresponding to the first three exons of *KANSL1.*

**Web Resources**

ClinVar: <https://www.ncbi.nlm.nih.gov/clinvar/>

gnomAD: <https://gnomad.broadinstitute.org/>

DGV: <http://dgv.tcag.ca/dgv/app/home>

Decipher: <https://www.deciphergenomics.org/>

UCSC Genome Browser: <https://genome.ucsc.edu/index.html>

Ensembl: <https://www.ensembl.org/index.html>

Primer3web: <https://primer3.ut.ee/>

OligoCalc: <http://biotools.nubic.northwestern.edu/OligoCalc.html>

**References**

1. Zou D, Wang L, Wen F, et al. Genotype‑phenotype analysis in Mowat‑Wilson syndrome associated with two novel and two recurrent ZEB2 variants. Exp Ther Med. 2020;20(6):263.
2. Wang K, Li M & Hakonarson H. ANNOVAR: functional annotation of genetic variants from high-throughput sequencing data. Nucleic Acids Research. 2010;38:e164–e164.
3. Koolen DA, Vissers LE, Pfundt R, et al. A new chromosome 17q21.31 microdeletion syndrome associated with a common inversion polymorphism. Nat Genet. 2006;38,:999–1001.
4. Koolen DA, Kramer JM, Neveling K, et al. Mutations in the chromatin modifier gene KANSL1 cause the 17q21.31 microdeletion syndrome. Nat Genet. 2012;44:639–641.
5. Zollino M, Orteschi D, Murdolo M, et al. Mutations in KANSL1 cause the 17q21.31 microdeletion syndrome phenotype. Nat Genet. 2012;44:636–638.
6. Koolen DA, Pfundt R, Linda K, et al. The Koolen-de Vries syndrome: a phenotypic comparison of patients with a 17q21.31 microdeletion versus a KANSL1 sequence variant. Eur J Hum Genet 2016;24:652–659.
7. Farnè M, Bernardini L, Capalbo A, et al. Koolen-de Vries syndrome in a 63-year-old woman: Report of the oldest patient and a review of the adult phenotype. *Am J Med Genet A*. 2022;188:692-707. doi: 10.1002/ajmg.a.62536.
